# Criterion Validity and Test-Retest Reliability of a Modified Version of the International Physical Activity Questionnaire–Short Form (IPAQ-SF) in Kidney Transplant Recipients

**DOI:** 10.3389/fresc.2022.808476

**Published:** 2022-02-10

**Authors:** Dario Kohlbrenner, Seraina von Moos, Gabriela Schmid-Mohler

**Affiliations:** ^1^Faculty of Medicine, University of Zurich, Zurich, Switzerland; ^2^Department of Pulmonology, University Hospital Zurich, Zurich, Switzerland; ^3^Division of Nephrology, University Hospital Zurich, Zurich, Switzerland; ^4^Center of Clinical Nursing Science, University Hospital Zurich, Zurich, Switzerland

**Keywords:** psychometric properties, clinimetrics, kidney transplantation, physical activity, accelerometry

## Abstract

**Introduction:**

Accelerometry, the clinically valued standard of physical activity monitoring, has limited acceptance in transplantation rehabilitation; therefore, the International Physical Activity Questionnaire (IPAQ) self-report instrument is widely used. However, while the IPAQ's repeatability is good, its criterion validity is unsatisfactory. We hypothesized that adding a concise oral introduction would help overcome this shortfall.

**Materials and Methods:**

This is a secondary analysis of a RCT in a sample of kidney transplant recipients that underwent observational follow-up. We assessed criterion validity of our modified version of the four-item IPAQ–Short Form (mIPAQ–SF) *via* Pearson, and test-retest reliability *via* intraclass correlation coefficients. The main difference in the new version is an oral pre-measurement introduction to the questionnaire's concepts. We compared our results with those of published studies.

**Results:**

Post-kidney-transplantation data of 92 patients were analyzed. Across the four IPAQ-SF/mIPAQ–SF items, values of correlations between mIPAQ-SF responses and accelerometry records ranged from 0.07 (min in vigorous activity) to 0.35 (min in moderate activity) for criterion validity, and from 0.19 (days with moderate activity) to 0.58 (min in moderate activity) for test-retest reliability.

**Discussion:**

Regarding moderate-to-vigorous physical activity, mIPAQ-SF self-reports' correlations to accelerometry records improved considerably on those of the IPAQ-SF (r = 0.18 vs. r = 0.33), i.e., improved criterion validity. We therefore conclude that a pre-measurement oral explanation of key IPAQ-SF/mIPAQ concepts enhances criterion validity regarding self-reported moderate-to-vigorous physical activity.

## Introduction

Physical activity (PA) monitoring has gained increasing consideration in recent years. Related research has achieved substantial findings, e.g., specifying the impact of regular PA vs. sedentary behavior in healthy and clinical populations (1, 2). Building on the available evidence, the World Health Organization (WHO) published an action plan with the goal of reducing the global prevalence of physical inactivity in adults first by 10%, then by 15%, respectively by 2025 and 2030 (3). Recognizing the benefits of sufficient PA regarding obesity, diabetes, hypertension and cardiovascular disease (3), the WHO now recommends age group-specific periods to spend in moderate-to-vigorous PA (MVPA) each week (4). These MVPA recommendations have been transferred to kidney transplant recipients (KTRs) with high cardiovascular risk (5, 6).

However, this group's adherence to PA recommendations tends to be poor (7). Both before and after transplantation, compared with healthy subjects, KTRs show low mean levels of PA (8). With age, their PA decreases to levels below those of other chronic disease groups (8). Therefore, ongoing support to help patients implement sufficient activity into their daily lives is now standard in post-transplantation clinical management.

Additionally, structured post-transplantation rehabilitation programmes have been developed to prevent, arrest or reverse pre-existing PA impairment. In many cases, such programmes can effectively halt the development of frailty and peripheral muscle dysfunction, both of which deteriorate health-related quality-of-life and increase mortality (6, 9).

Naturally, tailoring PA counseling to transplant populations requires adequate standardized monitoring. The gold standard of PA monitoring is accelerometry (7). However, for reasons thought to include poor acceptability to patients, device cost, difficulties with data extraction, the need for specially trained staff and the required seven-day period to collect reliable data (10), accelerometry has not yet become popular in clinical practice.

As an alternative, self-reporting questionnaires asking patients to rate their PA over a specified recall period may be used. Such questionnaires may be completed *via* a short interview format and are easily applicable across a broad range of clinical settings.

One widely-used example is the International Physical Activity Questionnaire (IPAQ). Also available as an easily applicable short-form questionnaire (IPAQ-SF), the IPAQ is designed to capture self-reported PA and sedentary behavior over either the last seven days or a hypothetical normal week (11). Translations into several languages allow broad international applicability (11). Furthermore, it can provide separate, detailed assessments of PA and sedentary time (i.e., covering 10-min segments and various intensities of PA) (11). This last feature makes the IPAQ appealing for transplantation rehabilitation settings, as it allows healthcare practitioners to target their feedback to specific intensity categories.

Problematically, despite the IPAQ's broad acceptance, its psychometric properties are limited. Specifically, while its test-retest reliability (repeatability) is good (11, 12), its criterion validity (correlation with known accurate measures, e.g., accelerometry) is poor (13). I.e., while repetitions of the test yield similar results, those results are not valid. This is particularly true regarding MVPA, which is widely overestimated (14). It is hypothesized that this results from a combination of social desirability and recall biases (15, 16). As older adults tend to perform their higher intensity PA in an unstructured manner their recall bias is generally more pronounced (17). As patients receive transplantation through a broad distribution of ages, they require a concise questionnaire that works similarly well for younger and older patients.

Rather than attempting to replace the IPAQ, we hypothesized that modifying the existing version would improve its accuracy without endangering its widespread acceptance. Accordingly, we made only minor changes to the questionnaire, but added a pre-measurement oral introduction to key concepts, plus descriptions clarifying the PA intensities asked for in the various items. We further hypothesized that this would reduce the respondents' recall bias, thereby enhancing their responses' accuracy (15, 17).

Our objectives were a) to assess the criterion validity and test-retest reliability of our modified version of the IPAQ-SF (mIPAQ-SF) in a sample of KTRs and b) to compare the results to those previously reported for the IPAQ-SF across healthy and other clinical populations. We hypothesized that, compared to the traditional IPAQ-SF, the mIPAQ-SF would show increased criterion validity, with test-retest reliability remaining similar.

## Materials and Methods

### Design

This study includes a secondary analysis of data from an RCT investigating the effects of a self-management program on weight management immediately after kidney transplantation, including three study visits (i.e., 2–6 weeks, 8 months and 12 months after kidney transplantation) (18). Patients received no further intervention between months 8 and 12, were in a stable condition, and showed little variation between their PA levels (18).

### Setting

The study was carried out at the University Hospital of Zurich. It was conducted in accordance with the declaration of Helsinki and all subjects provided written informed consent. The Ethics Committee of the Canton of Zurich approved the study (EK-ZH-NR: 2011-0411), which was registered on www.ClinicalTrials.gov (NCT02282124).

### Population

The sample was drawn from the population of patients who received kidney transplantation at our center from May 2012 to February 2018 (94 months). Data were collected over the entire period. Patients screened for eligibility were adults (i.e., 18 years or older) who had received a new kidney transplantation (18). We included patients with a complete data set, namely, for whom at least 4 days of accelerometry data were available at month 8 and 12, as well as complete mIPAQ-SF data at month 8 and 12.

### Sampling

The sample used for this analysis showed baseline characteristics similar to those of the Swiss KTR cohort study (19); therefore, it is considered generalizable to the Swiss KTR population.

### Data Collection

#### Modified IPAQ-SF

Self-reported PA was assessed *via* the mIPAQ-SF at months 8 and 12 after renal transplantation. The mIPAQ-SF assessed the number of days on which patients performed vigorous activity, moderate activity and walking. Then, the usual time per day spent in each specific activity was assessed (as reported in 10 min bouts). Aiming to enhance the original IPAQ's test-retest reliability and criterion validity, we modified the questionnaire's introduction (see Supplementary Material).

This began by introducing the patients to the mIPAQ-SF's conceptual framework, i.e., orally explaining the four levels of intensity (vigorous, moderate, walking and sitting) in a fully structured manner, along with criteria for bodily indicators of these levels (e.g., changes in breathing, heart rate and sweating) and examples of typical activities characterizing each intensity. Whereas, the original IPAQ-SF provided only breathing criteria (e.g., moderate: breathing somewhat harder than normal; vigorous: breathing much harder than normal), we added criteria for heart rate (i.e., moderate: pulse slightly faster; vigorous: pulse substantially faster) and perspiration (i.e., moderate: moderate perspiration; vigorous: profuse perspiration) (see Supplementary Material). Following this oral introduction, patients were given time to reflect on their activity levels over the previous 7 days. Thereafter, the mIPAQ-SF items were assessed by a registered nurse in a structured interview format.

#### Accelerometry

Accelerometry data on step counts and intensity levels were recorded *via* the ankle-worn StepWatch 3 Activity Monitor (Orthocare Innovations, Seattle, WA, USA) at 8 and 12 months after RT. The StepWatch 3 is validated in rehabilitation settings and has shown acceptable accuracy in individuals with slow walking speed and short stride (20). Following published guidelines, PA was categorized into three intensity levels: low (1–30 steps/min), moderate (31–80 steps/min) and vigorous (80 or more steps/min) (21). Patients were instructed to wear the device on their left ankle for a period of 7 days. Wearing time was only interrupted while taking showers. As proposed in best practice guidelines, accelerometry was only considered valid for analysis if wearing time was at least 4 days with at least 22 h of wearing time per day (10, 22).

### Data Analysis

In accordance with the Qualitative Attributes of PA (QAPA) checklist (23) and using accelerometry as the gold standard, Pearson correlation coefficients were used to investigate the mIPAQ-SF criterion validity regarding study visit data from month 12 (24). Intraclass correlation coefficients of type 3,1 (ICC_3,1_) were used to investigate the mIPAQ-SF's test-retest reliability between the study visits at months 8 and 12 (24). Reporting of test-retest reliability was conducted in accordance with the Guidelines for Reporting Reliability and Agreement Studies (GRAAS) (25), and the QAPA checklist (23).

Criterion validity was reported in accordance with the QAPA checklist (23). Considering MVPA's importance in clinical practice and health prevention settings (4), as it is not directly assessed by the mIPAQ-SF, we gauged validity and reliability based on its calculated value (i.e., summing the minutes spent in moderate and vigorous activity). We did the same for total physical activity (TPA), including walking and MVPA, which were also calculated (12). Only positive correlations were present in this analysis. In accordance with published recommendations, they were divided into five classifications: negligible (0 ≤ r ≤ 0.29), low (0.3 ≤ r ≤ 0.49), moderate (0.5 ≤ r ≤ 0.69), high (0.7 ≤ r ≤ 0.89) and very high (0.9 ≤ r ≤ 1) correlation (26). In accordance with published recommendations, ICCs were divided into five classifications: poor (0 ≤ ICC ≤ 0.20), fair (0.21 ≤ ICC ≤ 0.40), moderate (0.41 ≤ ICC ≤ 0.60), strong (0.61 ≤ ICC ≤ 0.80) and near complete (0.81 ≤ ICC ≤ 1) agreement (27). To visualize agreement between the mIPAQ-SF domains regarding average minutes of PA per day and accelerometry measurements, as well as regarding test-retest reliability, we used Bland-Altman Plots. These depict agreement between two measurement methods by plotting the differences between individual data points and their means (28, 29). In addition, we reported Bland-Altman statistics, i.e., mean differences and 95% limits of agreement (i.e., 1.96 SD) to allow conclusions on the magnitude of bias in the measurements and agreement.

Unless otherwise stated, reported results are shown as means (with SDs) or medians (and interquartile ranges). Significance level was set at *p* ≤ 0.05.

Statistical analyses were performed using R version 4.0.3 (R Core Team 2021, R Foundation for Statistical Computing, Vienna, Austria).

### Procedure

Patients were asked to wear accelerometers for the seven days before their study visits in months 8 and 12 post-transplantation. At each study visit they were also asked to complete the mIPAQ-SF in a structured interview format by a trained study nurse.

## Results

### Study Participants

Of the 123 patients participating in the study, 92 supplied complete accelerometry and mIPAQ-SF data for their follow-up visits. The sample consisted mainly of male (65%) patients with a median age of 55 (45–62) years. Baseline characteristics are presented in (Table 1); further sample information is published elsewhere (18). Detailed values of the data for each measurement time point is provided in (Table 2).

**Table 1:** Patient characteristics (*N* = 92).

**Variables**	**Value**
Age, years (median, interquartile range)	52 (45–62)
Sex, male/female (numbers, %)	60/32 (65/35)
Body-Mass Index, kg/m^2^ (median, interquartile range)	25.2 (22.4–27.7)
Steps per day, *n* (median, interquartile range)	5,196 (3,518–6,569)

*kg/m^2^, kilogram per squaremeter*.

**Table 2:** Values of the accelerometer and mIPAQ-SF measurements at 8 and 12 months.

**Intensity**	**8 Months**	**12 Months**
**Accelerometer**		
Minutes low	1,049 (979.9–1,095.3)	1,069.3 (983.4–1,135.6)
Minutes moderate	105.4 (75–131.3)	97.6 (70.5–126.1)
Minutes vigorous	23.1 (12.2–35.5)	20.7 (12.9–32.75)
Minutes moderate to vigorous (MVPA)	129 (89.2–164.5)	119 (79.3–157.3)
Days moderate	7 (7–7)	7 (7–7)
Days vigorous	5 (3–6)	5 (3–6)
**mIPAQ-SF**		
Minutes seated	270 (150–360)	270 (180–450)
Minutes moderate	60 (30–97.5)	60 (25–120)
Minutes vigorous	30 (0–60)	0 (0–60)
Minutes moderate to vigorous (MVPA)	105 (46.3–150)	75 (38.5–156)
Days moderate	5 (2–7)	5 (2–7)
Days vigorous	0 (0–2)	0 (0–2)

*Values are median (interquartile range). mIPAQ-SF, modified International Physical Activity Questionnaire – Short Form*.

### Criterion Validity of the Modified IPAQ-SF

For numbers of minutes spent in vigorous activity (per day) we found a negligible correlation between mIPAQ-SF reports and accelerometry records [r (95% CI) = 0.07 (−0.13, 0.26), *p* = 0.52]. We found low correlations between number of minutes spent in MPA [r (95% CI) = 0.35 (0.16, 0.5), *p* < 0.001], MVPA [r (95% CI) = 0.33 (0.14, 0.45), *p* = 0.001], and TPA [r (95% CI) = 0.34 (0.15, 0.51), *p* < 0.001].

Regarding correlations between number of days with episodes of at least 10 min of different levels of activity, we found negligible correlation between mIPAQ-SF reported and accelerometry recorded number of days with bouts of at least 10 min of vigorous activity [r (95% CI) = 0.15 (−0.05, 0.33), *p* = 0.15], the number of days with bouts of at least 10 min of moderate activity [r (95% CI) = 0.11 (−0.10, 0.30), *p* = 0.31], and the number of days with bouts of at least 10 min spent walking [r (95% CI) = 0.19 (−0.01, 0.37), *p* = 0.07]. Correlations for validity are displayed in (Table 3).

**Table 3:** Pearson correlation coefficient table for validity between accelerometry and the mIPAQ-SF.

**Variables**	**r (95% CI)**	***p*-value**
Minutes vigorous	0.07 (−0.13, 0.26)	0.52
Minutes moderate	0.35 (0.16, 0.51)	<0.001
Minutes moderate to vigorous (MVPA)	0.33 (0.14, 0.45)	0.001
Minutes low-moderate-vigorous (TPA)	0.34 (0.15, 0.51)	<0.001
Days vigorous	0.15 (−0.05, 0.33)	0.15
Days moderate	0.11 (−0.10, 0.30)	0.31
Days walking	0.19 (−0.01, 0.37)	0.07

*mIPAQ-SF, modified International Physical Activity Questionnaire – Short Form*.

**Figure 1:**
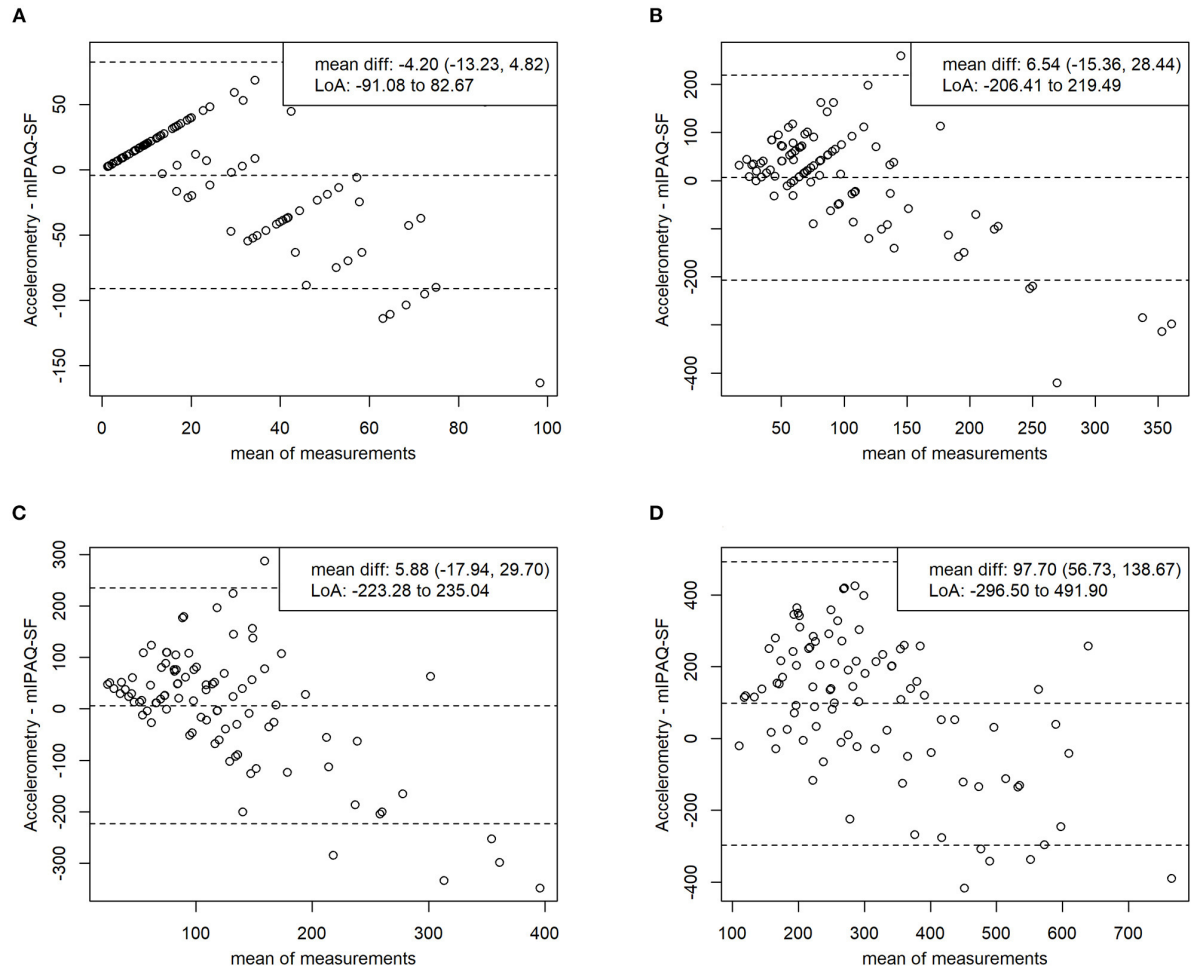
Bland-altman plots validity. Comparing average minutes intensive per day **(A)**, average minutes moderate per day **(B)**, average minutes moderate to vigorous per day **(C)**, average minutes total physical activity **(D)** between mIPAQ-SF and accelerometry. Accelerometry – mIPAQ-SF: difference between methods; mIPAQ-SF: modified International Physical Activity Questionnaire – Short Form; mean diff: mean difference with 95% confidence interval; LoA, limits of agreement (1.96 SD).

The Bland-Altman analysis depicting criterion validity regarding average minutes of TPA per week shows a mean difference (95% CI) of 97.70 (56.73, 138.67) minutes, indicating significant underestimation of TPA per week in the mIPAQ-SF. Limits of agreement were from −296.50 to 491.90 min. Regarding average minutes per day, the mean differences (95% CI) were −4.20 (−13.23, 4.82) min for vigorous PA, 6.54 (−15.36, 28.44) min for moderate PA, and 5.88 (−17.94, 29.70) min for MVPA, indicating no significant over- or underestimation in the mIPAQ-SF. Limits of agreement were from −91.08 to 82.67 min for vigorous PA, from −206.41 to 219.49 min for moderate PA, and from −223.28 to 235.04 min for MVPA. Scattering of data gets wider with increasing number of minutes reported for all items. The results of the Bland-Altman analysis are displayed as Bland-Altman plots in (Figure 1).

### Test-Retest Reliability of the Modified IPAQ-SF

Comparing the two follow-up tests (conducted in months 8 and 12), we found moderate agreement between reported numbers of minutes spent in vigorous activity [ICC_3,1_ (95% CI) = 0.58 (0.47, 0.67), *p* < 0.001], walking [ICC_3,1_ (95% CI) = 0.52 (0.40, 0.62), *p* < 0.001], TPA [ICC_3,1_ (95% CI) = 0.48 (0.36, 0.59), *p* < 0.001], and sitting [ICC_3,1_ (95% CI) = 0.55 (0.43, 0.64), *p* < 0.001]. We also found fair agreement between numbers of minutes spent in moderate activity [ICC_3,1_ (95% CI) = 0.23 (0.09, 0.37), *p* = 0.005], and between the two reports' numbers of minutes spent in MVPA [ICC_3,1_ (95% CI) = 0.28 (0.13, 0.41), *p* < 0.001]. Correlations for reliability are displayed in (Table 4).

**Table 4:** ICC table for reliability of the mIPAQ-SF between 8- and 12-month follow-up.

**Variables**	**ICC_**3, 1**_ (95% CI)**	***p*-value**
Minutes vigorous	0.58 (0.47, 0.67)	<0.001
Minutes walking	0.52 (0.40, 0.62)	<0.001
Minutes walking-moderate-vigorous (TPA)	0.48 (0.36, 0.59)	<0.001
Minutes sitting	0.55 (0.43, 0.64)	<0.001
Minutes moderate	0.23 (0.09, 0.37)	0.005
Minutes moderate to vigorous (MVPA)	0.28 (0.13, 0.41)	<0.001
Days vigorous	0.34 (0.20, 0.46)	<0.001
Days moderate	0.19 (0.04, 0.03)	0.017
Days walking	0.25 (0.10, 0.38)	0.003

*mIPAQ-SF, modified International Physical Activity Questionnaire – Short Form; ICC, intraclass correlation coefficient*.

**Figure 2:**
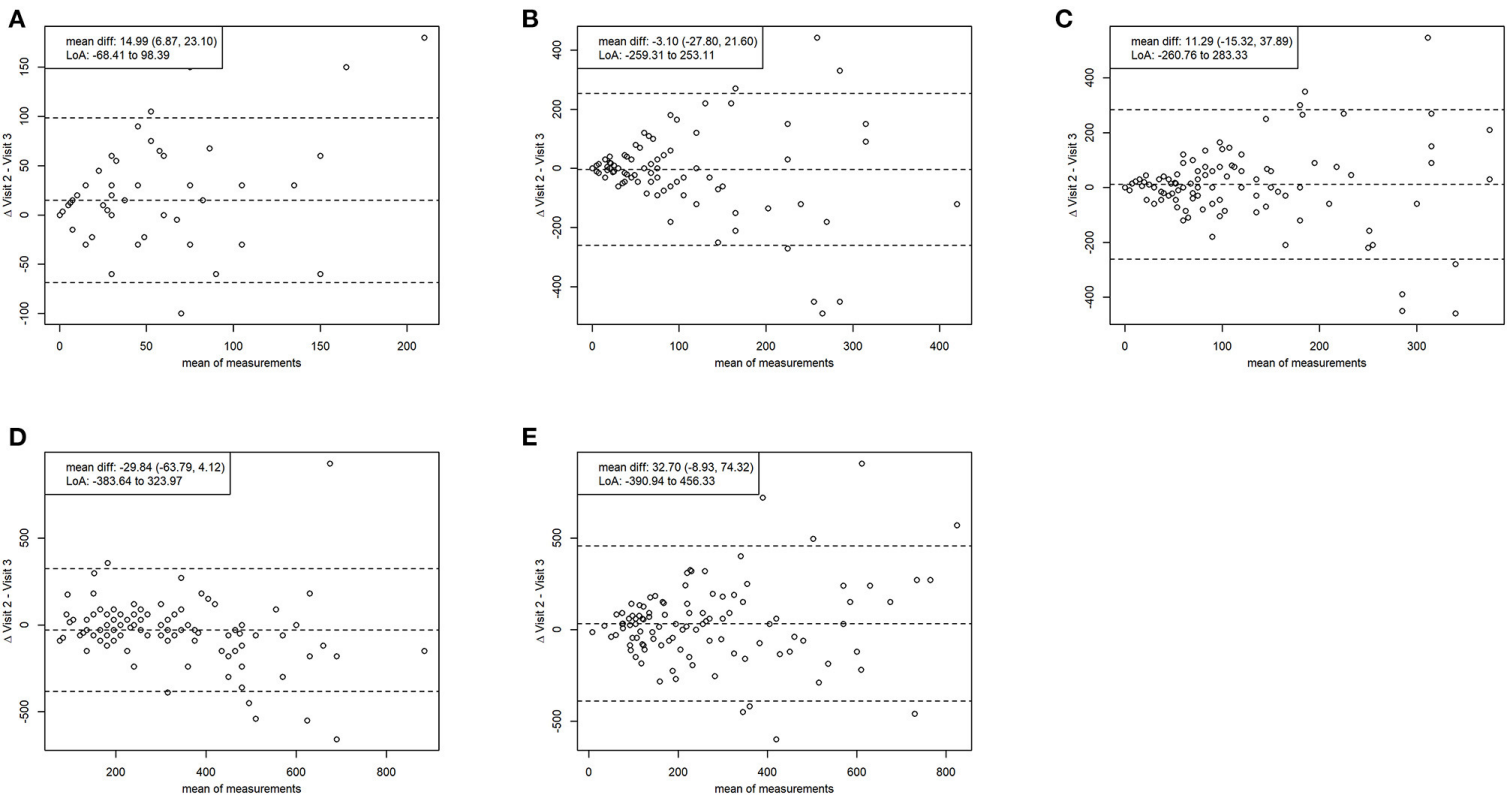
Bland-altman plots reliability. Comparing average minutes intensive per day **(A)**, average minutes moderate per day **(B)**, average minutes moderate to vigorous per day **(C)**, average minutes sitting per day **(D)**, average minutes total physical activity **(E)** between the two measurement time points. Mean diff., mean difference with 95% confidence interval; LoA, limits of agreement (1.96 SD).

The Bland-Altman analysis depicting test-retest reliability for average minutes per day of TPA shows a mean difference (95% CI) of 32.70 (−8.93, 74.32) min, indicating no significant over- or underestimation between the visits. Limits of agreement were from −390.94 to 456.33 min. However, for average minutes per day of vigorous PA, the mean difference (95% CI) falls to 14.99 (6.87, 23.10) min, indicating significant overestimation at visit 2. Limits of agreement were from −68.41 to 98.39 min. Regarding mean inter-test differences (95% CI) between average reported daily times were −3.10 (−27.80, 21.60) min for moderate PA, 11.29 (−15.32, 37.89) min for MVPA, and −29.84 (−63.79, 4.12) min for sitting, all indicating no significant over- or underestimation between the visits. Limits of agreement were from −259.31 to 253.11 min for moderate PA, from −260.76 to 283.32 min for MVPA, and from −383.64 to 323.97 min for sitting. The agreement does not diminish with increasing numbers in reporting. The results of the Bland-Altman analysis are displayed as Bland-Altman plots in (Figure 2).

## Discussion

This study investigated the criterion validity and test-retest reliability of a modified version of the IPAQ–SF, a physical activity (PA) self-report instrument, in a sample of (*n* = 92) KTR. The mIPAQ–SF version adds a structured oral introduction explaining the instrument's key concepts. This and other minor modifications were aimed at enhancing the earlier version's low criterion validity as well as its test-retest reliability.

According to WHO recommendations, time spent in MVPA is particularly important for health-related outcomes (4). In fact, combining data on these two activity levels into a single score—MVPA—is a technique commonly used in clinical practice. Regarding patient-reported data, it has been suggested that MVPA also offers greater recall accuracy than either of its separate domains. Comparing patient-reported mIPAQ-SF data on MVPA to accelerometry records, we found significant low correlation, i.e., criterion validity. Regarding test-retest reliability, the evidence indicated fair agreement in this domain.

In our analysis, comparing the mIPAQ-SF's criterion validity to that of the IPAQ-SF, MVPA was the domain whose validity improved most (r = 0.33 vs. r = 0.18) (13). In addition, no significant bias was shown in the Bland-Altman analysis, indicating improved validity as compared to the IPAQ-SF (14). However, agreement is highest in individuals with low level of MVPA. As physical activity acts as an independent predictor of weight gain and glucose tolerance in kidney transplant recipients (30, 31), this is an important finding with practical implications for the transplant setting. Regarding the modified instrument's increased validity regarding MVPA, the mIPAQ-SF's combination of low cost, convenience and especially validity make it a promising alternative to accelerometry in clinical practice (4). Considering test-retest reliability, our results indicate fair agreement regarding MVPA. This was lower than previously reported (12, 13).

For moderate PA, compared with accelerometry results, the mIPAQ–SF showed improved criterion validity (r = 0.35 vs. r = 0.30) (12, 13). However, compared to the traditional form, it showed lower validity regarding minutes spent in vigorous activity (r = 0.07 vs. r = 0.31). In addition, Bland-Altman analysis of test-retest reliability showed significant bias. We can only speculate on the underlying reasons for this. However, as intensity thresholds commonly implemented in accelerometry reflect healthy population data, we hypothesize that patients with chronic disease may experience, and accordingly classify, vigorous activity differently. Our increased use of bodily symptoms to determine activity levels might have increased this discrepancy.

The strength of this study is its investigation of the criterion validity of two identically scaled activity count-based measures (i.e., minutes spent at a defined intensity) in their respective questionnaires as compared to the gold standard. Furthermore, we were able to analyse all of the questionnaires' subdomains except the validity of items regarding time spent sitting, as the accelerometer used could not record this.

This study also has notable limitations. The mIPAQ-SF requests information on PA during a “usual week,” aiming to obtain an individually generalizable conclusion on PA levels. In contrast, accelerometry recordings may have recorded unstructured and intermittent PA that is unusual for the individual participant, not covering one of the “usual weeks” and therefore not delivering generalizable information. Accordingly, our study might have underestimated the mIPAQ-SF's validity. Secondly, despite the participant's apparent lack of changes regarding either PA or demographics between their visits at months 8 and 12 post transplantation (18), other changes may have occurred, influencing factors related to PA and not covered by our assessments. Last, the accelerometer used lacks validation studies regarding the different intensity thresholds implemented. To the best of our knowledge, existing validation literature focuses exclusively on the validity in step count.

The development of well-tolerated customer-grade PA monitors is a very dynamic and rapidly developing field. There remains the possibility that accurate, robust, well-tolerable, and reasonably priced accelerometers become available in due time. However, we still believe that activity questionnaires remain a rapid and easily applicable tool in clinical rehabilitation. It remains to be studied if combining objective and subjective activity monitoring would add valuable information for clinicians.

In conclusion, compared to the original IPAQ–SF, the mIPAQ–SF may be more accurate and therefore more suitable for patients when MVPA is of special interest—as is the case in KTR. However, it remains to be considered that validity, although improved with the mIPAQ-SF, remains mostly in the low spectrum. The mIPAQ-SF is less suitable for populations in which vigorous PA is of interest; and further investigation in larger samples would be necessary before broad application. Accordingly, clinicians should always consider if the application of an accelerometer is feasible and appropriate in the first place.

Nevertheless, considering the IPAQ-SF's importance as a PA outcome measure, the modified version's improved validity regarding MVPA and sufficient test-retest reliability are promising developments. As a further implication for future research, we would recommend investigating the mIPAQ-SF in other populations.

## Data Availability Statement

The raw data supporting the conclusions of this article will be made available by the authors, without undue reservation.

## Ethics Statement

The studies involving human participants were reviewed and approved by the Ethics Committee of the Canton of Zurich, Switzerland. The patients/participants provided their written informed consent to participate in this study.

## Author Contributions

GS-M: design and data collection. DK: data analysis and manuscript writing. DK, GS-M, and SM: interpretation and manuscript revision. All authors contributed to the article and approved the submitted version.

## Funding

This study was registered at www.ClinicalTrials.gov (NCT02282124).

## Conflict of Interest

The authors declare that the research was conducted in the absence of any commercial or financial relationships that could be construed as a potential conflict of interest.

## Publisher's Note

All claims expressed in this article are solely those of the authors and do not necessarily represent those of their affiliated organizations, or those of the publisher, the editors and the reviewers. Any product that may be evaluated in this article, or claim that may be made by its manufacturer, is not guaranteed or endorsed by the publisher.
