# Supplementary material for: Criterion Validity and Test-Retest Reliability of a Modified Version of the International Physical Activity Questionnaire–Short Form (IPAQ-SF) in Kidney Transplant Recipients
Source: Front Rehabil Sci. 2022 Feb 10;3:808476. doi: 10.3389/fresc.2022.808476 (PMC9397873; doi:10.3389/fresc.2022.808476)
Supplement: Supplementary file 1 [file Data_Sheet_1.docx]

# INTERNATIONAL PHYSICAL ACTIVITY QUESTIONNAIRES IPAQ: SHORT LAST 7 DAYS SELF-ADMINISTERED FORMAT

**FOR USE WITH YOUNG AND MIDDLE-AGED ADULTS**

The International Physical Activity Questionnaires (IPAQ) comprises a set of 4 questionnaires. Long (5 activity domains asked independently) and short (4 generic items) versions for use by either telephone or self-administered methods are available. The purpose of the questionnaires is to provide common instruments that can be used to obtain internationally comparable data on healthrelated physical activity.

## Background on IPAQ

The development of an international measure for physical activity commenced in Geneva in 1998 and was followed by extensive reliability and validity testing undertaken in 12 countries (14 sites) across 6 continents during 2000. The final results suggest that these measures have acceptable measurement properties for use in many settings and in different languages. IPAQ is suitable for use in regional, national and international monitoring and surveillance systems and for use in research projects and public health program planning and evaluation. International collaboration on IPAQ is on-going and an international prevalence study is under development.

## Using IPAQ

Worldwide use of the IPAQ instruments for monitoring and research purposes is encouraged. It is strongly recommended, to ensure data quality and comparability and to facilitate the development of an international database on health-related physical activity, that

- no changes be made to the order or wording of the questions as this will affect the psychometric properties of the instruments,
- if additional questions on physical activity are needed they should follow the IPAQ items,
- translations are undertaken using the prescribed back translation methods (see website)
- new translated versions of IPAQ be made available to others via the web site to avoid duplication of effort and different versions in the same language,
- a copy of IPAQ data from representative samples at national, state or regional level be provided to the IPAQ data storage center for future collaborative use (with permission) by those who contribute.

## More Information

Two scientific publications presenting the methods and the pooled results from the IPAQ reliability and validity study are due out in 2002.

More detailed information on the IPAQ process, the research methods used in the development of the IPAQ instruments, the use of IPAQ, the published papers and abstracts and the on-going international collaboration is available on the IPAQ web-site. **www.ipaq.ki.se**

This is the final SHORT LAST 7 DAYS SELF-ADMINISTERED version of IPAQ from the 2000/01 Reliability and Validity Study. Completed May 2001.

**INTERNATIONAL PHYSICAL ACTIVITY QUESTIONNAIRE**

**IPAQ: SHORT LAST 7 DAYS SELF-ADMINISTERED FORMAT**

**FOR USE WITH YOUNG AND MIDDLE-AGED ADULTS**

*NOTE*: EXAMPLES OF ACTIVITIES MAY BE REPLACED BY CULTURALLY RELEVANT EXAMPLES WITH THE SAME METS VALUES (SEE AINSWORTH *ET AL*., 2000).

# INTERNATIONAL PHYSICAL ACTIVITY QUESTIONNAIRE

We are interested in finding out about the kinds of physical activities that people do as part of their everyday lives. This is part of a large study being conducted in many countries around the world. Your answers will help us to understand how active we are compared with people in other countries.

The questions are about the time you spent being physically active in the last 7 days. They include questions about activities you do at work, as part of your house and yard work, to get from place to place, and in your spare time for recreation, exercise or sport.

Your answers are important.

**Please answer each question even if you do not consider yourself to be an active person.**

**THANK YOU FOR PARTICIPATING.**

In answering the following questions,

♦ **vigorous** physical activities refer to activities that take hard physical effort and make you breathe much harder that normal.

♦ **moderate** activities refer to activities that take moderate physical effort and make you breathe somewhat harder that normal.

| 1a. | During the last 7 days, on how many days did you do **vigorous** physical activities like heavy lifting, digging, aerobics, or fast bicycling? |
| --- | --- |
|  |  |
|  | Think about *only* those physical activities that you did for at least 10 minutes at a time. |
| 2a.                3a. | **________ days per week**  1b. How much time in total did you usually spend on one of those days doing  vigorous physical activities*?*    **_____ hours ______ minutes or none**  Again, think *only* about those physical activities that you did for at least 10 minutes at a time. During the last 7 days, on how many days did you do **moderate** physical activities like carrying light loads, bicycling at a regular pace, or doubles tennis? Do not include walking.  **________ days per week**  2b. How much time in total did you usually  spend on one of those days doing moderate physical activities*?*    **_____ hours ______ minutes or none**  During the last 7 days, on how many days did you **walk** for at least 10 minutes at a time? This includes walking at work and at home, walking to travel from place to place, and any other walking that you did solely for recreation, sport, exercise or leisure.  **________ days per week**  3b. How much time in total did you usually spend walking on one of those days*?*    **_____ hours ______ minutes or none** |

**The last question is about the time you spent sitting on weekdays while at work, at home, while doing course work and during leisure time. This includes time spent sitting at a desk, visiting friends, reading traveling on a bus or sitting or lying down to watch television**.

4. During the last 7 days, how much time in total did you usually spend *sitting* on a **week day?**

**____ hours ______ minutes**

**This is the end of questionnaire, thank you for participating.**
