# Supplementary material for: Criterion Validity and Test-Retest Reliability of a Modified Version of the International Physical Activity Questionnaire–Short Form (IPAQ-SF) in Kidney Transplant Recipients
Source: Front Rehabil Sci. 2022 Feb 10;3:808476. doi: 10.3389/fresc.2022.808476 (PMC9397873; doi:10.3389/fresc.2022.808476)
Supplement: Supplementary file 2 [file Table_1.docx]

**IPAQ Modification Supplement**

| **Vigorous-intensity activity** | Your heart rate is substantially faster  You breathe much harder than normal  Sweat  Examples:  Jogging  Bicycling fast or uphill  Swimming fast  Aerobic  Soccer game or other fast ball games  Tennis single  Carrying heavy loads |
| --- | --- |
|  |  |
| **Moderate-intensity activity** | Your heart rate is slightly faster  You breathe somewhat harder than normal  Somewhat sweat    Examples:  Walking briskly  Bicycling at moderate speed and at ground level  Swimming at moderate speed or Aquafit  Tennis doubles  Cleaning heavy (washing windows and cleaning floors, mowing the lawn)  Climbing stairs  Carrying light loads |
|  |  |
| **Walking** | Examples:  Walking at normal pace or slowly  Standing light work (cooking, washing dishes, ironing) |
|  |  |
| **Sitting** | Examples:  Sitting at work, school, at home, public transportation or car  Sitting with friends, at the table, watching TV, reading |
